# Antimicrobial Resistance Profile and ExPEC Virulence Potential in Commensal *Escherichia coli* of Multiple Sources

**DOI:** 10.3390/antibiotics10040351

**Published:** 2021-03-26

**Authors:** Elisa Massella, Federica Giacometti, Paolo Bonilauri, Cameron J. Reid, Steven P. Djordjevic, Giuseppe Merialdi, Cristina Bacci, Laura Fiorentini, Paola Massi, Lia Bardasi, Silva Rubini, Federica Savini, Andrea Serraino, Silvia Piva

**Affiliations:** 1 Department of Veterinary Medical Sciences, Faculty of Veterinary Medicine, University of Bologna, Ozzano Emilia, 40064 Bologna, Italy; elisa.massella@libero.it (E.M.); federica.savini3@unibo.it (F.S.); andrea.serraino@unibo.it (A.S.); silvia.piva@unibo.it (S.P.); 2 Experimental Zooprophylactic Institute of Lombardy and Emilia Romagna, 42124 Reggio Emilia, Italy; paolo.bonilauri@izsler.it; 3 The iThree Institute, University of Technology Sydney, Ultimo, NSW 2007, Australia; cameron.reid@uts.edu.au (C.J.R.); steven.djordjevic@uts.edu.au (S.P.D.); 4 Experimental Zooprophylactic Institute of Lombardy and Emilia Romagna, 40127 Bologna, Italy; giuseppe.merialdi@izsler.it (G.M.); lia.bardasi@izsler.it (L.B.); 5 Department of Veterinary Medical Sciences, Faculty of Veterinary Medicine, University of Parma, 43126 Parma, Italy; cristina.bacci@unipr.it; 6 Experimental Zooprophylactic Institute of Lombardy and Emilia Romagna, 47122 Forlì, Italy; laura.fiorentini@izsler.it (L.F.); paola.massi@izsler.it (P.M.); 7 Experimental Zooprophylactic Institute of Lombardy and Emilia Romagna, 44124 Ferrara, Italy; silva.rubini@izsler.it

**Keywords:** commensal *Escherichia coli*, indicator organism, antimicrobial resistance, resistance trends, quinolone resistance, ExPEC virulence potential, food safety

## Abstract

We recently described the genetic antimicrobial resistance and virulence profile of a collection of 279 commensal *E. coli* of food-producing animal (FPA), pet, wildlife and human origin. Phenotypic antimicrobial resistance (AMR) and the role of commensal *E. coli* as reservoir of extra-intestinal pathogenic *Escherichia coli* (ExPEC) virulence-associated genes (VAGs) or as potential ExPEC pathogens were evaluated. The most common phenotypic resistance was to tetracycline (76/279, 27.24%), sulfamethoxazole/trimethoprim (73/279, 26.16%), streptomycin and sulfisoxazole (71/279, 25.45% both) among the overall collection. Poultry and rabbit were the sources mostly associated to AMR, with a significant resistance rate (*p* > 0.01) to quinolones, streptomycin, sulphonamides, tetracycline and, only for poultry, to ampicillin and chloramphenicol. Finally, rabbit was the source mostly associated to colistin resistance. Different pandemic (ST69/69*, ST95, ST131) and emerging (ST10/ST10*, ST23, ST58, ST117, ST405, ST648) ExPEC sequence types (STs) were identified among the collection, especially in poultry source. Both ST groups carried high number of ExPEC VAGs (pandemic ExPEC STs, mean = 8.92; emerging ExPEC STs, mean = 6.43) and showed phenotypic resistance to different antimicrobials (pandemic ExPEC STs, mean = 2.23; emerging ExPEC STs, mean = 2.43), suggesting their role as potential ExPEC pathogens. Variable phenotypic resistance and ExPEC VAG distribution was also observed in uncommon ExPEC lineages, suggesting commensal flora as a potential reservoir of virulence (mean = 3.80) and antimicrobial resistance (mean = 1.69) determinants.

## 1. Introduction

*Escherichia coli* represents a commensal colonizer of human and animal gastrointestinal microbiota [1] and it is the most frequently isolated Gram-negative pathogen impacting human health [2]. *E. coli* is a worrisome public health threat due to its outstanding variability in pathotypes (enteropathogenic *E. coli*—EPEC, enterohaemorrhagic *E. coli*—EHEC, enterotoxigenic *E. coli*—ETEC, enteroaggregative *E. coli*—EAEC, enteroinvasive *E. coli*—EIEC, diffusely adherent *E. coli*—DAEC, uropathogenic *E. coli*—UPEC, meningitis associated *E. coli*—MNEC, sepsis associated *E. coli*—SEPEC), multiple infection sites (intestinal or extraintestinal), clinical symptomatology [1] and concerning antimicrobial resistance (AMR) profile, especially to carbapenemase and extended-spectrum beta-lactams [3,4]. The different *E. coli* pathotypes have been associated to specific phylogroups, each one showing distinct phylogenetic relatedness [5,6].

In particular, ExPEC represents one of the most common causes of bloodstream infections and community/hospital associated urinary tract infections worldwide [7,8]). They are also responsible for other extraintestinal diseases, being an important cause of neonatal meningitis [1]. Multiresistant ExPEC strains constitute ongoing healthcare concern and are associated with an increase in infection severity, treatment failure, hospitalisations and mortality, with growing costs for health care [2]. ExPEC mostly belonged to phylogroup B2 and, to a lesser extent, to phylogroup D [9,10].

It has been hypothesised that ExPEC are opportunistic pathogens. They may occupy a niche in human and animal intestinal microflora, showing their virulence potential while colonising extraintestinal sites [11]. Discrimination with molecular epidemiological approaches between potential ExPEC and commensals could be challenging to afford, because of the share of large genomic fractions and different VAGs, involved in common fitness function [12,13]. Therefore, ExPEC could be hidden among commensal flora, which could also represent a reservoir of virulence genes for these pathogens.

Commensal *E. coli* is also recognised as an AMR barometer among Gram-negative bacteria, due to its ubiquity and genomic plasticity. Indeed, *E. coli* represents the prevailing organism able to grow in aerobic conditions in the gastrointestinal microbiota of warm-blooded animals [14] and is also an environmentally adapted bacterium [15]. Its genomic plasticity allows constant and efficient exchange of genomic fractions, including genes conferring resistance to antimicrobials, with other enteric bacteria and the environment. Hence, AMR data acquired from *E. coli* indicator is considered representative of the overall bacterial population [16].

AMR epidemiological surveillance is an important tool, signaling changes in current bacterial AMR trends [17]. The data gathered from epidemiological surveillance allow the implementation of preventive and control strategies, including suitable antimicrobial stewardship programs, therapeutic guidelines and infection control policies [18].

*E. coli* indicator is routinely used in European AMR monitoring to oversee AMR in FPAs and related food since 2014 [16]. Attention has been focused on specific FPA categories (poultry, swine, turkey and bovine), because of their high population size and important meat demand (http://www.fao.org/faostat/en/, last accessed 30 June 2020). Additionally, overall antimicrobial consumption in the European Union (EU) is mostly associated to livestock, hence exposed to high antimicrobial selective pressure [19].

FPAs could play a primary role in AMR diffusion through direct/indirect contact with humans, related food and environmental (land and water) manure contamination [20,21,22,23,24]. Furthermore, they could represent an important AMR reservoir, where foodborne pathogens may obtain new antimicrobial resistance genes and develop novel resistance patterns [25].

In the last few decades AMR sources other than livestock have proved to be particularly concerning.

AMR bacteria and genes have been identified in human related environments (livestock, companion animals, animal and non-animal origin food) as well as remote ecosystems (wildlife), where antimicrobial selective pressure is supposed to be absent [23,26,27,28,29,30]. These data suggest the complexity of AMR dynamics, influenced by the thriving antimicrobial resistance gene (ARG) bacterial trade and the interconnection between different ecosystems.

AMR arises from selective pressure induced by antimicrobial treatments. Resistant bacteria and genes can be gathered, maintained and transmitted through horizontal gene transfer (HGT) within any environmental niche, determining the diffusion of novel AMR profiles in the overall bacterial population [31]. The complex interconnection between different ecosystems, sharing common habitats and water sources, is responsible for the wide AMR geographical distribution. Water and soil faecal contamination plays a primary role in AMR spread, establishing a link between various environments [32]. Further investigations are needed to achieve a better comprehension of AMR dynamics, focusing on the identification of potential AMR sources and transmission paths that are posing a risk to human health.

In our recent paper [33] we described the population structure, ARG and VAG carriage in a collection of 279 *E. coli* indicator of animal (livestock, pets, wild animals), food (vegetable and animal origin products) and human origin collected in Italy. *E. coli* strains were grouped in 12 sources (dairy, beef, wild boar, rabbit, poultry, swine, companion animal, vegetable, fishery, mollusc, wild animal and human), according to their origin. Concerning genetic AMR profile have been identified, including to highest priority critically important antimicrobials (HP-CIAs). Different typical ExPEC VAGs and pandemic and emerging ExPEC STs were observed among the overall collection.

Considering these previous findings, the current study aims to investigate (i) phenotypic antimicrobial resistance profile of the collection and concordance with genetic AMR profile previously identified, in order to establish the potential AMR risk associated with animals (livestock, companion animals, wildlife), food and human, (ii) ExPEC virulence potential of commensal *E. coli,* association with concerning AMR profile and their role of ExPEC VAG reservoir, (iii) phylogroup distribution, considering the revisited phylotyping method proposed by Clermont et al., (2013) [6], for further epidemiological evaluations.

## 2. Results

### 2.1. Occurrence of E. coli Strains in Animal, Food and Human Samples

*E. coli* was identified in 169/415 (40.72%) samples analysed. In particular, *E. coli* was detected in all companion animal (12/12) and swine (6/6) samples and in 25/27 (92.59%) human faeces analysed (Table 1). High *E. coli* occurrence was observed in poultry (25/33, 75.76%), rabbit (10/14, 71.43%), wild boar (22/31, 70.97%) and beef (21/34, 61.76%) samples. Fishery and vegetables were the sources with the lowest occurrence of *E. coli*, identified in only 24/94 (25.53%) and 24/164 (14.63%) samples, respectively. It was not possible to establish *E. coli* occurrence in mollusc, dairy and wild animal sources, since related strains were provided from the Experimental Institute for Zooprophylaxis in Lombardy and Emilia Romagna.

### 2.2. Antimicrobial Resistance Phenotypes

Among the 279 *E. coli* strains, 107 (38.35%) showed resistance to at least one antimicrobial and 79 (28.32%) were MDR (Table 2). The most common phenotypic resistances were to tetracycline (76/279, 27.24%), sulfamethoxazole/trimethoprim (73/279, 26.16%), streptomycin and sulfisoxazole (71/279, 25.45% both), ampicillin (66/279, 23.66%), followed by nalidixic acid (48/279, 17.20%), enrofloxacin (44/279, 15.77%), chloramphenicol (23/279, 8.24%) and gentamicin (11/279, 3.94%). Lastly, 3GC (ceftiofur and ceftazidime) and colistin resistances were observed in 6/279 (2.15% each) strains. The most common antimicrobials implicated in MDR were tetracycline, sulphonamides, streptomycin and ampicillin.

Four out of 279 strains (1.43%) were designated as ESBL producers. Notably, all ESBL producers were MDR, including to other HP-CIAs (nalidixic acid, enrofloxacin, colistin).

Considering the different sources investigated, the mean number of resistance was: rabbit, 6; poultry, 4; dairy, companion animal, swine and human, 2 each; beef, fishery and wild animal, 1 each. The lowest resistance (mean ≤ 1) was observed in mollusc, vegetable and wild boar sources, with 4, 2 and 1 resistant strains, respectively. Notably, rabbit and poultry were the sources displaying the most extensive AMR, with 23/23 (100%) and 23/25 (92%) strains resistant to at least one antimicrobial agent, respectively. Most of the strains (22/23, 95.7% in rabbit; 16/25, 64% in poultry) were MDR from 3 up to 8 different antimicrobial classes. Furthermore, rabbit was the niche mainly associated to colistin resistance (3/6).

3-GC resistance was mainly reported in dairy (2) and beef (2) strains. Interestingly, the dairy source carried the highest number of ESBL producers (2/4).

Wild animal, vegetable, fishery and companion animal niches generally displayed low resistance when compared to the other sources. MDR strains were detected with a lesser extent (companion animal, 3/12, 25%; wild animal, 6/25, 24%; fishery, 3/24, 12.5%; vegetable, 2/24, 8.33%), though resistance to HP-CIAs (Qs, 3-GCs) was observed in 6 strains (5 wild animals, 1 companion animal).

Notably, one ESBL producer was associated to wildlife (wild animal and wild boar sources).

**Figure 1:**
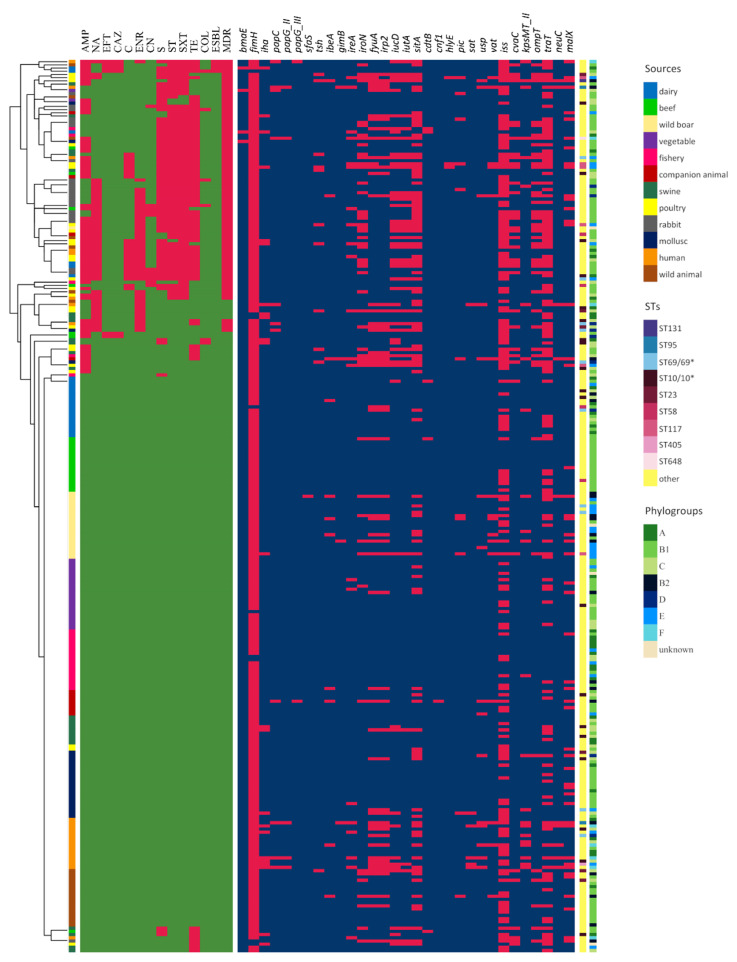
Heat map depicting phenotypic AMR and carriage of typical ExPEC VAGs in the strain collection. The dendogram on the left represents clustering of *E. coli* isolates according to their phenotypic AMR profile. Presence of phenotypic AMR and carriage of typical ExPEC VAGs are shown in red, with a green (for phenotypic AMR) or blue (for ExPEC VAGs) square indicating their absence. Additional strain information is provided in column 1- 3 and includes: source (column beside the dendogram), ST (first column after the heatmap) and phylogroup (second column after the heatmap). Sources, STs and phylogroups are colour-coded as described in the legend.

Wild boars and molluscs revealed the lowest antimicrobial resistance among all the collection. Five strains showed resistance to at least one antimicrobial agent (1/22, 4.54%, wild boar; 4/25, 16%, mollusc), of which 4 (wild boar, 1; mollusc, 3) were MDR. Resistance to HP-CIAs was detected in 1 wild board and in 1 mollusc FQ resistant strains. The detailed phenotypic antimicrobial profile of the overall collection is shown in Figure 1.

The phenotypic AMR pattern identified among the collection was generally concordant with the genetic AMR profile previously identified (Table 3). Discrepancies only occurred with nalidixic acid/enrofloxacin (6 strains), chloramphenicol (1 strain) and colistin (1 strain) antimicrobials.

### 2.3. Phylogroups

Seven different phylogroups (A, B1, B2, C, D, E and F) were identified in the collection, as described in Table 4. Phylogroup B1 was the most common (127/279, 45.52%), followed by C (43/279, 15.41%), A (38/279, 13.62%), E (25/279, 8.96%), B2 (20/279, 7.17%), F (13/279, 4.66%) and D (9/279, 3.23%).

Four strains were designated as unknown since it was not possible to assign them to any phylogroup.

One specific phylotype was usually representative in each source despite variability in phylogroup distribution and abundance. Phylogroup B1 was mainly associated to beef (22/24, 91.67%), rabbit (20/23, 86.96%), wild animal (18/25, 72%), mollusc (15/25, 60%), vegetable (14/24, 58.3%), companion animal (6/12, 50%) and dairy (9/25, 36%) sources. Phylogroup A was most common in fishery (10/24, 41.67%), meanwhile phylogroup C was mostly represented in poultry (8/25, 32%). Swine strains were equally associated to phylogroup A (8/25, 32%), B1 (8/25, 32%) and C (8/25, 32%). Interestingly, “pathogen” phylogroups were mainly associated to human (F, 7; E, 3; B2/D, 2 both) and wild boar (E, 11; B2, 6) strains.

Notably, strains showing resistance to the highest number of antimicrobials (≥5) were mainly associated to “non pathogen” phylogroups (B1, 33; A–C, 6 both) and with a lesser extent to the “pathogen” ones (F, 6; E, 3; D, 2).

**Figure 2:**
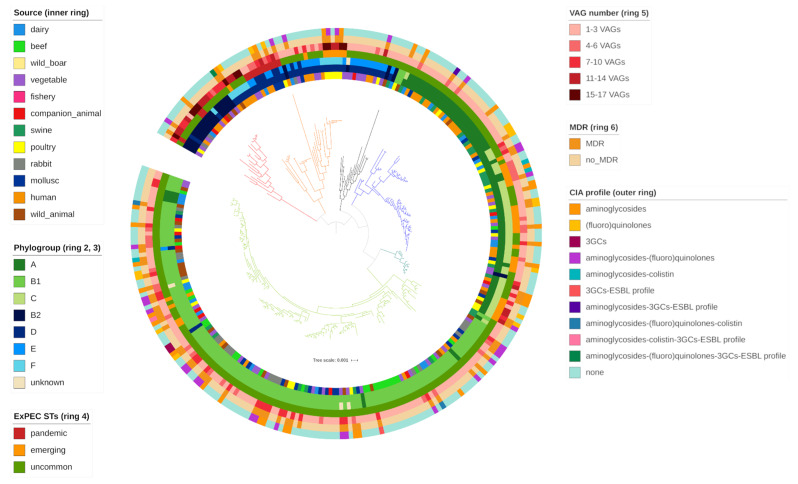
A mid-point rooted, maximum-likelihood phylogenetic tree of 279 commensal *E. coli* included in the study. The phylogenetic tree is rearranged from the original one, whose comprehensive description is available in the previous publication [33]. Branches are coloured by clade and subclade (red= clade 1, subclade 1; orange= clade 1, subclade 2; black= clade2, subclade 1; blue= clade 2, subclade 2; sea green= clade 2, subclade 3; light green= clade 2, subclade 4). Source (inner ring), phylogroup according to Clermont quadruplex PCR (ring 2), phylogroup according to in silico method (ring 3), emergent/pandemic/uncommon ST (ring 4), VAG number (ring 5), MDR profile (ring 6), HP-CIA resistance profile (outer ring) are annotated according to the legend. A comprehensive description of the phylogenetic tree is available in the previous publication [33].

Comparison between molecular and in silico phylotyping data showed important concordance. Only 4 strains, properly assigned to a phylotype with ARIBA [34], were designated as unknown with the quadruplex PCR method (Figure 2).

### 2.4. Expec Virulence Potential

Thirty-one typical ExPEC VAGs were identified among the overall collection (Table 6). All strains carried between 1 and 17 VAGs (Figure 2), assembled in variable virulence profiles (Figure 1). Among the collection, VAG occurrence was as follow: *fimH* (270/279; 96.77%), *iss* (162/279; 58.06%), *traT* (125/279; 44.8%), *sitA* (100/279; 35.84%), *fyuA* (55/279; 19.71%), *irp2/iucD* (53/279; 18.90%), *iroN/malX* (50/279; 17.92%), *ompT* (45/279; 16.13%), *iutA* (41/279; 14.70%), *cvaC* (37/279; 13.26%), *iha/kpsMT* (23/279; 8.24%), *ireA* (21/279; 7.52%), *vat* (16/279; 5.73%), *usp* (15/279; 5.38%), *papC/tsh/ibeA/pic* (11/279; 3.94%), *neuC* (10/279; 3.58%), papG (7/279; 2.51%), *sat* (6/279; 2.15%), *cdtB* (5/279; 1.79%), *bmaE/gimB* (4/279; 1.43%), *hlyE* (3/279; 1.08%), *sfaS/cnf1* (1/279; 0.36%).

Thirteen strains belonged to pandemic ExPEC STs, namely 10 ST69/69* (dairy, 1; wild boar, 2; poultry, 1; mollusc, 3; human, 3), 2 ST95 (human) and 1 ST131 (poultry). As expected, pandemic ExPEC STs showed the highest number of VAGs (mean = 8.92) and AMR phenotypes (mean = 2.23).

Interestingly, 7/13 (53.85%) pandemic ExPEC STs were associated to AMR, of which 6 were multidrug-resistant. Notably, ST131 showed the highest number of resistance to 9 antimicrobials. HP-CIA resistance was observed in 3 FQ resistant ST69 strains of poultry, mollusc and human origin. All pandemic ExPEC STs belonged to typical ExPEC phylogroups (B2, 3; D, 5; E, 5).

Thirty-seven emerging ExPEC STs were detected in different sources, namely 19 ST10/ST10* (dairy, 3; beef, 1; vegetable, 1; companion animal, 1; swine, 7; poultry, 2; mollusc, 2; human, 2), 6 ST23 (poultry, 3; mollusc, 1; wild animal, 2), 4 ST58 (dairy, 1; beef, 2; companion animal, 1), 6 ST117 (1 wild boar; 5, poultry), 1 ST405 (human) and 1 ST648 (wild animal). Most of the strains were associated to commensal phylotypes (A, 4; B1, 4; C, 21), with the exception of ST117 (5, F; 1F), ST405 (E) and ST648 (F) belonging to typical ExPEC phylogroups. VAG carriage and AMR phenotype mean were 6.43 and 2.43 respectively. MDR was observed in 14/37 (37.84%) strains. Notably 10 Q and 1 colistin resistant strains were identified.

The remaining 229 strains carried the lowest AMR phenotypes (mean = 1.69) and VAG number (mean = 3.80) among the 3 groups identified. Variable phylogroup distribution (A = 34; B1 = 123; B2 = 17; C = 22; D = 4; E = 18; F = 7) was observed. Fifty-three out of 59 MDR strains detected belonged to commensal phylogroups. Uncommon ExPEC STs showed the highest HP-CIA resistance, with 35 Q, 4 colistin resistant strains and 4 ESBL producers.

Pandemic, emerging and uncommon ExPEC STs showed variable distribution of genes coding for the 5 functional categories involved in ExPEC pathogenesis (Table 5).

Interestingly, eleven strains carried genes coding for all 5 functional categories. Threestrains belonged to typical ExPEC STs (ST131, 1; ST95, 2) and phylotypes (B2, 9; D, 1; E, 1), meanwhile 8 strains were associated to uncommon ExPEC STs (ST141, ST680, ST1170, ST80, ST706*, ST420*, ST136, ST372) but typical ExPEC phylogroups (B2, 6; D, 1; F, 1). MDR was observed in ST131 (1), ST95 (2), ST706* (1) strains.

Complete phenotypic AMR and genetic virulence profile, STs and phylogroup distribution among the collection are reported in Supplementary Materials (Tables S2–S4).

### 2.5. Statistical Results

Statistical analysis identified poultry and rabbit as the most important AMR sources among the collection. In these niches, the occurrence of resistance to at least one molecule and of MDR was statistically significant (*p*-value < 0.01) if compared to the other sources. Poultry and rabbit strains exhibited significantly higher (*p*-value < 0.01) resistant rates to Qs, streptomycin, sulfisoxazole, sulfamethoxazole/trimethoprim and tetracyline than the remaining collection. Furthermore, significant ampicillin and chloramphenicol resistance was observed in poultry (*p*-value < 0.01).

Considering the other sources, no significant resistance was detected, except for tetracycline resistance in swine strains (*p* < 0.01).

Important differences in phylogroup richness and eveness were identified among the sources (Table 4), with the highest diversities in human (H′ = 1.814; D′ = 0.850) and poultry (H′ = 1.691; D′ = 0.817) and the lowest in rabbit (H′ = 0.470; D′ = 0.245) and beef (H′ = 0.345; D′ = 0.163). Association between specific phylogroups and phenotypic antimicrobial patterns was investigated. Statistical analysis identified a significant association for ampicillin resistance—E/F phylotype and sulfisoxazole resistance—F phylotype (*p* < 0.05 both).

## 3. Discussion

AMR is a complex and ever-changing phenomenon, whose dynamics are still not completely understood. Epidemiological surveillance is an essential weapon against AMR threat, allowing to define key point sources where AMR could develop, evolve and spread. AMR monitoring in different environments represents a fundamental cornerstone for epidemiological evaluation and preventive/control measure implementation [18]. Indeed, multiple sources (animals, agriculture, human, food) have been investigated as a potential reservoir of AMR in the last decades [23,26,28,29,30,35].

Our previous study aimed to provide an overview of AMR genetic profile currently circulating in *E. coli* indicator in Italy. *E. coli* from different origins have been considered, including environments playing a well documented (FPAs, food, pets, human) or emerging (wildlife) role in AMR dynamics. Greater emphasis was given to livestock due to their important epidemiological role in AMR spread through human contact [36,37], environmental manure contamination [21,38] and related foodstuff [23,39,40,41,42]. FPAs may play an important role in the development of new antimicrobial resistance patterns, increasing the AMR gene pool available for foodborne pathogens [25].

Particular attention was given to HP-CIAs (including extended-spectrum cephalosporins, quinolones and colistin), recognised as the last treatment option for serious human infections with a possible FPA/food origin [7].

Antimicrobial susceptibility testing results were interpreted using ECOFF in order to perform an epidemiological evaluation about phenotypic AMR circulating in *E. coli* indicator of different origins.

In the present paper, wild type (WT) strains, without an acquired phenotypically detectable resistance mechanism, were defined as “susceptible”. Non-WT strains, related to acquired or mutational resistance to antimicrobials, were addressed as “resistant”.

Unfortunately, ECOFFs were not available for all the molecules tested, hence clinical breakpoints were adopted. Clinical breakpoints represented predictors of clinical success of antimicrobial treatments, but could not afford evaluation related to emergence and evolution in bacterial resistance profiles [43,44]. Nevertheless, they could provide worthwhile information about consolidated resistance patterns in the bacterial population. Changing in consolidated clinical resistant profiles could indirectly suggest the emergence of new resistance patterns among bacteria.

### 3.1. Tetracycline, Sulfonamide, Streptomycin and Ampicillin Resistance Is the Most Common among the Overall Collection

The most common phenotypic resistance was to tetracycline, sulfonamides (sulfisoxazole and sulfamethoxazole/trimethoprim), streptomycin and ampicillin among FPAs/food, companion animal, wildlife and humans.

The wide diffusion of resistance to tetracycline, sulfonamides and ampicillin is probably attributable to antimicrobial usage in different human and animal sectors in Europe. Sulfonamides and tetracycline represent the most common treatment in FPAs (swine, poultry, beef, rabbit and dairy), as well as ampicillin in FPAs (swine, poultry, beef, rabbit and dairy), companion animals and human [45]. In particular, the zootechnical system is associated to the highest antimicrobial consumption in Europe [19], turning livestock into an important reservoir of AMR bacteria and genes. Indeed, the high frequency of resistance to sulfonamide-ampicillin-tetracycline in swine, poultry, beef, rabbit and dairy strains was generally congruent with AMR profiles previously reported in Europe [16,46,47,48,49,50,51,52]. The same resistance pattern (sulfonamide-ampicillin-tetracycline resistance) was also observed in the strictly connected human environment, linked in turn with companion animals. High ampicillin resistance rate probably derived from the wide use of these molecules in human and pets [45], whereas sulfonamide and tetracycline resistance presumably originated from agricultural settings. Sulfonamide-ampicillin-tetracycline resistance has also been previously observed in human and pets strains [53,54].

Interestingly, streptomycin resistance was the third-highest phenotypic resistance (together with sulfisoxazole) (24.45%) among all the collection and it was identified in at least one strain of each source. These data are generally consistent with previous studies in animal and human [32,47,55,56,57,58,59]. Exceptions are represented by pig, poultry and beef, usually showing low level of aminoglycoside resistance. Streptomycin resistance is inconsistent with the seldom use of aminoglycosides in both human and animal therapeutic treatment [45,60], which account for low gentamycin resistance among the collection (3.94%) instead. So far, the evidence leads to the hypothesis of streptomycin resistance wide diffusion being referable to ARGs co-selection mechanisms, mediated by multiresistant plasmids [61] and transposons [62,63]. Additionally, streptomycin is one of the oldest antimicrobials discovered in 1943 [64] and its past use could explain the active resistance circulation detected nowadays.

### 3.2. The Potential Role of Aquaculture, Vegetable and Wildlife as AMR Source Sentinels

Generally, aquaculture, vegetable and wildlife (wild animal and wild boar) are sources slightly connected to AMR. In aquaculture (mollusc and fishery) antimicrobial treatments are rare [65], meanwhile vegetable and wildlife are not directly exposed to antimicrobials. The consistency of AMR profile identified in these niches with those observed in the remaining collection (livestock, human, companion animals) suggested an important AMR environmental pollution from settings highly associated to antimicrobial use, in line with findings reported by Giacometti et al., (2021) [66]. Water and environment faecal contamination appear the most important path allowing ARGs dissemination in ecosystems where antimicrobial use is rare or absent [32]. Irrigational water/manure [57,67] and sewage/runoff from land [68,69,70] have already been implicated in AMR emergence in aquaculture and vegetable products, respectively. Likewise, remoteness of area, zootechnical activity and human proximity represented the most important parameters influencing AMR occurrence in wildlife [71,72].

Interestingly, resistance to sulfonamide-ampicillin-tetracycline in aquaculture [56,73], vegetable [56,57,74] and wildlife [75,76] has already been reported in different countries suggesting the wide extension of AMR diffusion problem in outlying environments.

Considering all these assumptions, aquaculture, wildlife and vegetable could represent important AMR source-sentinel, giving useful information about specific AMR spread and the degree of AMR environmental contamination. However, the low *E. coli* indicator occurrence in fishery, mollusc and vegetable sources could suggest wildlife as the most promising and concrete AMR spread indicator. Additionally, low *E. coli* occurrence indicates fishery, mollusc and vegetable as low risk food-sources associated to transmission of potential pathogenic *E. coli* to human, when compared to swine, poultry and rabbit niches, where *E. coli* was frequently identified.

### 3.3. Phenotypic Q Resistance Is the Most Common Among HP-Cias

HP-CIA resistance represents a worrisome event, associated with significant morbility/mortality and treatment alternatives reduction, and has required considerable control during food production [7]. Qs are included in the HP-CIA list and represent the last treatment options in serious *Salmonella* spp. and *E. coli* infections [7]. Notably, Q resistance was mainly observed in FPAs, with a lesser extent in human and wildlife strains, and not detected in companion animals and vegetable.

Considering FPAs, the most common HP-CIA resistance was to Qs (21.05%, nalidixic acid; 18.71%, enrofloxacin), identified in all the livestock categories but in fishery source. The highest FQ resistance was observed in rabbit (nalidixic acid, 15/25; enrofloxacin, 13/25) and poultry (nalidixic acid, 13/25; enrofloxacin, 11/25) strains, presumably because of the common use of these antimicrobials in related breeding systems [65]. Resistance rates in these niches were significantly higher (*p* < 0.01) than those detected in the other FPA categories, where Q detection was notably lower. Our data are in accordance with previous studies reporting generally high Q resistance in poultry and rabbit in contrast to other livestock [47,48,49,52,77,78,79].

As for livestock, the highest HP-CIA resistance observed in human and wildlife was to Qs (24% and 15%, respectively). Notably, our data showed considerable higher Q resistance in human, as well as in wildlife, than those reported in previous studies [53,75,76,80,81,82,83].

These findings are particularly concerning, considering the increasing trend of Q resistance in human clinical *E. coli* in Europe, with Italy as the country showing the highest resistance [8].

### 3.4. Poultry and Rabbit Are the Sources Mainly Associated to AMR

AMR was mainly associated to livestock, with poultry and rabbit as the sources showing significant higher resistant rate (*p* < 0.01 or *p* < 0.05) to most molecules (Qs, streptomycin, sulfisoxazole, sulfametoxazole/trimethoprim, ampicillin and chloramphenicol in poultry), when compared to other niches.

These data are not surprising considering poultry intensive breeding systems are characterised by notably higher population density compared to the other FPAs categories [84]. Population size is strictly interconnected with antimicrobial usage, often characterised by collective treatments, and therefore influencing antimicrobial selective pressure [85]. Indeed, poultry have been frequently associated with generally higher AMR than other livestock in European countries [86]. These considerations could be extended also to rabbit farming.

Poultry AMR profile was generally in accordance with previous studies reporting tetracycline, Qs (nalidixic acid and enrofloxacin), ampicillin, sulfamethoxazole, trimethoprim resistance as frequently detected, meanwhile those to 3GCs and colistin were overall low. Contrary to our results, resistance to chloramphenicol was variable, meanwhile aminoglycoside resistance was generally rare [48,49,50,78,79]. Our findings in rabbit were in accordance with the seldom AMR data available in rabbit breeding system, reporting resistance to tetracyclines, ampicillin, aminoglycosides (streptomycin, gentamicin), Qs (ciprofloxacin, nalidixic acid) and sulfonamides (sulfadiazine, sulfamethoxazole/trimethoprim) as common phenotypic AMR profile [47,87,88,89]. Interestingly, rabbit was the source mostly associated to colistin resistance. Recent studies described colistin resistance in rabbit farms [88], also in Italy, where colistin treatment is reported as common [90]. In our knowledge, this is the first report identifying phenotypic colistin resistance associated to rabbit meat, highlighting the possible involvement of rabbit breeding system in colistin resistance diffusion through the food-chain.

Interestingly, poultry and rabbit resistant strains were mostly isolated from food products of animal origin (poultry meat, 13; rabbit meat, 10) and with a lesser extent from animals (poultry, faeces, 2; rabbit, intestine, 5). The low number of samples analysed could not afford a proper evaluation of AMR epidemiological risk associated to animal and related foodstuff. However, our data could suggest an important involvement of the food chain in AMR transmission, presumably from zootechnical settings up to consumers. This hypothesis is supported by different studies, suggesting poultry as a feasible origin of AMR clinical *E. coli* identified in human [91,92,93,94]. The scarce European data on AMR in rabbit breeding and foodstuff [47,87,88,89] encouraged further investigations to better elucidate the role of rabbit in AMR transmission to human.

### 3.5. Phenotypic Pattern Is Generally Concordant with Genotypic AMR Profile

The phenotypic AMR pattern identified among the collection was generally concordant with the genetic AMR profile previously identified (Table 5). Discrepancies only occurred with Qs (6 strains), chloramphenicol (1 strain) and colistin (1 strain) antimicrobials. Qs resistant strains carrying *qnr* genes generally provided low level of resistance [95], but in our collection *qnrS1* gene (3) did not present with the expected phenotype. Their importance is mostly associated to the selection of specific chromosomal mutations, favoring the emergence of strain with higher FQ resistance [96].

FQ inefficacy is frequently associated to mutations in the quinolone resistance-determining region (QRDR: *gyrA/gyrB* and *parC/parE*), coding for the drug targeting enzymes DNA gyrase (GyrA/GyrB) and topoisomerase IV (ParC/ParE) subunits.

Generally, single or combined mutations in *gyrA*, *parC*, *parE* were associated with the phenotypic resistance profile. Interestingly, in some cases, substitutions Ala-56 → Thr in the ParC (ATC → ACC in *parC*) and Ile-355 → Thr in the ParE (ATC → ACC in *parE*) proteins, previously associated to resistant *E. coli* [97,98], were identified in nalidixic acid and enrofloxacin susceptible strains.

Chloramphenicol resistance was usually explained by the identification of genes coding for chloramphenicol acetyltransferases (*cat*) or specific chloramphenicol exporters (*cml*) [99]. Interestingly, *cat* and/or *cml* genes were not identified in one chloramphenicol resistant strain. Carriage of *mdfA* and *acrA-acrB-TolC* genes, coding for the aspecific multidrug transporter MdfA [100] and AcrAB-TolC [101], could explain the observed resistant phenotype.

In the present study, colistin resistance was associated to plasmid mediated *mcr1* gene, coding for a phosphoethanolamine transferase. This enzyme is responsible for a cationic modification of the LPS of the bacterial outer cell membrane, target of polymyxin antimicrobials [102].

One colistin susceptible strain carried Val-161 → Gly mutation in PmrB, associated to both polymyxin resistance and susceptibility [103,104,105]. *pmrB*, together with *pmrA* gene, coded for the PmrAB two-component system activation, typically involved in bacterial survival against cellular mediated immune response [106]. Mutations in these genes could potentially lead to resistance, changing lipopolysaccharide charge and reducing polymyxin attachment to the external surface of Gram-negative bacteria [107].

### 3.6. Important Phylogroup Variability Occurred among the Different Sources

Clermont revisited phylotyping method [6,108] identified up to 7 different phylotypes among the collection, with important variability among FPAs, pets, human and wildlife.

As expected, B1 (127/279; 45.52%), C (43/279; 15.41%) and A (38/279; 13.62%) were the most common phylogroups among all the collection and also in most sources. These phylotypes are considered “generalists” of multiple hosts and are commonly identified in the commensal population [109].

Notably, typical ExPEC phylotypes were mainly observed in wild boar (17/23, 73.91%; 11, E; 6, B2) and human (14/25, 56%; 7, F; 3, E; 2, B2; 2, D) sources. Our findings are generally in accordance with previous studies describing wild boar strains mostly belonging to B2 and D (including derivatives) [110,111,112] and D (including derivatives) as the second most common phylogroup in human commensal isolates worldwide [6].

Important variability in phylogroup distribution has been observed among sources (table), especially in human (H′ = 1.814; D = 0.850) and some FPA categories (poultry H′ = 1.691, D = 0,817; dairy: H′ = 1.485, D = 0.763; fishery: H′ = 1.457, D = 0.764) showing the highest phylogroup richness.

A proper comparison between phylogroup distribution in our collection and those reported in previous studies is hard to perform, due to the phylotyping method used and the multiple factors influencing phylogroup appearance.

Phylogroup determination is mostly performed according to Clermont scheme, targeting two genes (*chuA*, *yiaA*) and a DNA fragment (TSpE4.C2) [5]. This method allows to identify only the four main phylogroup A, B1, B2, D. The lack of proper C, E, F identification prevents detailed epidemiological evaluation of *E. coli* population genetics in different environments. Furthermore, important information regarding AMR and virulence potential associated to these minor phylogroups are lacking.

Differences in phylogroup distribution are mainly ascribable to geographical (location and climate) and host (diet, gut morphology, body mass) factors [113], explaining variable animals and human phylogroup identification in different studies [109,110,113,114,115,116,117,118,119,120].

Generally, human commensal *E. coli* are worldwide mostly associated to phylogroup A and D [6,114], meanwhile FPAs mostly belonged to phylogroup A and B1 [109,110]. Differences in wild animal phylotype distribution have been observed, with D and B1 as the most common in birds, A in wild rabbits and D and B2 in wild boars [110].

### 3.7. Clermont Quadruplex PCR Is a Valid Alternative to In Silico Phylotyping Technique

Molecular and in silico phylotyping data were compared, considering that C, E, F phylotypes, identified with the revisited Clermont scheme [108], would be included in the four main phylogroups (A, B1, B2, D) in the original triplex phylogroup assignment method [5].

Important concordance between results obtained with the two phylotyping techniques was observed. Only exceptions were represented by 4 strains designated as unknown with the quadruplex PCR method and properly assigned to a phylotype with ARIBA.

These findings suggest Clermont quadruplex PCR as a suitable alternative for phylogroup determination to in silico phylotyping. Indeed, quadruplex PCR implementation had contained costs, is time sparing and does not need specific and expensive technology for its implementation.

### 3.8. Commensal E. coli Conceal Potential Multiresistant Pandemic and Emerging Expec Pathogens

ExPEC are responsible for a majority of human extraintestinal infections worldwide. Actual ExPEC pathogenic potential is interconnected with VAG number [121], as with the expression of specific VFs involved in ExPEC pathogenesis. Indeed, studies in animal models of extraintestinal infection suggest VF profile as an in vivo virulence predictor [122,123].

Successful ExPEC pathogens must perform a range of functions during pathogenesis, namely adhesion/colonisation, host defence evasion, multiplication, tissue damage and diffusion [1]. Specific VFs are essential in each phase of pathogenesis and are generally divided in 5 functional categories [11]: (i) adhesins, important adherent factors promoting host cell contact, hence adhesion and colonisation [124]; (ii) invasins, mediating cell invasion into the host tissues [124]; (iii) iron acquisition systems, allowing iron uptake in low iron conditions (i.e., in host fluids and tissue) [125]; (iv) toxins, responsible of tissue lesion and promoter of local bacteria diffusion, cytotoxicity and insensitivity to neutrophils [126]; (v) protectins, structural components of the bacterial outer membrane involved in host defense evasion (including resistance to innate immunity and serum survival increase) [127].

Notably, 50 pandemic and emerging ExPEC lineages were identified in our collection. Both groups showed high number of typical ExPEC VAGs (pandemic ExPEC mean = 8.92; emerging ExPEC mean = 6.43), including those coding for adhesins (*fimH* and *papG*), invasins (*ibeA* and *gimB*), iron acquisition systems (*ireA*, *iroN*, *fyuA*, *irp2*, *iucD*, *iutA*, *sitA*), serum survival protectins (*iss* and *traT*) and toxins (*usp*, *vat*, *pic*, *sat*, *hlyE*).

ST131, ST95 and ST69/69* are members of the predominant clonal ExPEC group, with ST131 as the most common ExPEC lineage isolated worldwide [128].

B2 ST131 (1) and B2 ST95 (2) strains carried an outstanding number of VAGs (17 and 12 respectively), coding for VFs of all the functional categories involved in ExPEC pathogenesis.

Their genetic virulence pattern showed similarities to clinical ST131 and ST95 prototypic virulence profile [129], suggesting the virulence potential of these strains.

Notably, ST95 strains carried *papG_II*, a specific *papG* allele coding for the P pilus tip adhesin, responsible for UPEC adhesion in the urinary tract [130,131]. *papG_II* adhesin recognises glycolipid receptors located in the human kidney [132] and is frequently associated with pyelonephritis [133]. Also toxin genes (*usp* and *vat*), typically identified in UPEC [35,134,135,136], were observed in both ST131 and ST95 strains.

On the other side, ST69/69* strains showed significant variability in VAGs carriage (between 2 and 12) with rather different genetic traits compared to those commonly described in clinical strains [129]. For example, toxin genes (*cdtB*, *hlyD*, *cnf1*) were not identified, though they are commonly reported in this lineage [137].

The high number of strains carrying typical ExPEC VAGs is particularly worrisome. It has been hypothesised that ExPEC represent facultative pathogens. They may occupy a niche in human and animal intestinal microflora, showing their virulence potential while colonising extraintestinal sites [11]. Discrimination with molecular epidemiological approaches between potential ExPEC and commensals could be challenging to afford, because of the share of large genomic fractions and different VAGs, involved in common fitness function [11].

ExPEC could be hidden among commensal flora, arising and showing their virulence with favourable conditions occurrence. Further studies are needed to better investigate commensal *E. coli* virulence traits and to elucidate their role in ExPEC infections. Identification of commensal *E. coli* virulence profile could be useful to determine sources potentially associated to ExPEC transmission.

Additionally, the highest phenotypic AMR was observed in both pandemic (mean = 2.23; MDR = 46.15%) and emerging (mean = 2.43; MDR = 37.84%) STs. A possible reason could be the potential co-carriage of virulence and antimicrobial determinants on the same genetic platform (plasmids, transposons, integrons), co-mobilized under antimicrobial selective pressure [138]. A typical example is represented by F plasmid replicons that are considered the *E. coli* major virulence-associated plasmids, as well as multiple ARG carriers [139,140,141]. Interestingly, HP-CIA resistance was mainly described in uncommon ExPEC lineages, though this AMR profile is more likely identified in ExPEC STs [142].

### 3.9. E. coli Indicator Are Expec VAG Reservoir

Although uncommon ExPEC lineages group was associated to the lowest number of VAGs (mean = 3.80), the virulence profile showed extreme variability in VAG number (from 1 up to 15 VAGs) and presence of VFs related to different functional categories (from 1 up to 5). In our findings, uncommon ExPEC lineages seem unlikely associated to virulence potential. However, ExPEC genetic traits in the commensal population may be acquired from potential ExPEC pathogens, coexisting in the gastrointestinal tract. Therefore, commensal *E. coli* could represent a critical VAG reservoir, increasing virulent armament of potential ExPEC pathogen or allowing the acquisition of virulence traits in traditionally avirulent bacteria. A notable exception is represented by 8 strains, carrying genes coding for all functional category members. Phylogroup association (6 B2, 1 D, 1 F) and virulence profile were similar to those observed in B2 ST131 and B2 ST95 strains, suggesting that in some cases unconventional ExPEC lineages may display ExPEC potential.

### 3.10. Pandemic and Emerging Expec STs Mainly Belonged to Poultry Source

Variable ExPEC profile has been observed in various sources, with poultry as the niche mostly associated to ExPEC potential. Indeed 11/25 (44%) poultry (ST117, 5; ST23, 3; ST131, 1; ST69, 1; ST10, 1) strains belonged to emerging or pandemic ExPEC STs and carried high number of VAGs (poultry: mean = 11.73).

Poultry has been suspected to represent an ExPEC animal reservoir for humans, due to the close genetic relationship between human EXPEC and avian pathogenic *E. coli* (APEC). Indeed, ExPEC and APEC may share genome content, phylogeny and genetic virulence profile [143]. Additionally, experimental studies suggest APEC as potential pathogen in mammals, as well as human-derived ExPEC showing their virulence in avian animal models [144]. Concerningly, different studies identified potential ExPEC STs in poultry meat, suggesting its role as a vector of potential ExPEC pathogen to human [145,146].

Faeces contamination of carcasses during slaughtering procedures seems to be the most feasible path of ExPEC diffusion through the food chain [147,148] as APEC are assumed to coexist with commensal microflora in gastrointestinal tract of asyntomatic animals [149].

## 4. Materials and Methods

### 4.1. Samples Collection

This study analysed a collection of 279 commensal *E. coli*, arranged in 12 sources: poultry (n = 25), swine (n = 25), vegetable (n = 24), fishery (n = 24), mollusc (n = 25), wild animal (n = 25) and human (n = 25).

For bacterial strain gathering, a total of 433 samples of food, animal and human origin (beef, 33; wild boar, 31; vegetable, 164; fishery, 94; companion animal, 12; swine, 6; poultry, 33; rabbit, 14; human, 27) were assembled and processed in the period between 2010 and 2018. After analysis, 169 *E. coli* were collected (beef, 21; wild boar, 22; vegetable, 24; fishery, 24; companion animal, 12; swine, 6; poultry, 25; rabbit, 10; human, 25).

Additionally, 110 presumptive *E. coli* isolates were provided by the Department of Veterinary Medical Science—University of Bologna—Service of Food Safety (dairy, 25) and Experimental Institute for Zooprophylaxis in Lombardy and Emilia Romagna (beef, 3; rabbit, 13; swine, 19; mollusc, 25; wild animal, 25) and included in the study.

The strains retain their original name and the respective genome sequences are available in the National Center for Biotechnology Information (NCBI) (https://www.ncbi.nlm.nih.gov/, last accessed 30 Jane 2020). Accession numbers are listed in Supplementary Materials Tables S2–S4.

### 4.2. Genetic Features of the Collection

In our previous study an overall description of *E. coli* population structure, genetic virulence and antimicrobial resistance profile and phylogroups was described and herafter summarised. One hundred-eight out of 279 (38.71%) strains carried between 1 and 18 ARGs, with consistent variability in genetic resistance profile among sources. The most common ARGs were tetracycline resistance gene *tetA* (57/279, 20.43%), sulfonamide resistance gene *sul2* (45/279, 16.12%), penicillin resistance gene *bla*_TEM-1b_ (43/279, 15.41%) and streptomycin resistance genes *strA/B* (42/279; 15.10%) among the overall collection. In that study, genes and chromosomal point mutations conferring resistance to HP-CIAs were widely detected, including to quinolones (Qs) (*qnrS1*, *qnrB19*, *gyrA/parC/parE*), colistin (*mcr1, pmrB)* and 3^d^ generation cephalosporins (3GC) (*bla*_CMY-2_, *ampC*). Q resistant determinants were mainly observed in rabbit (17/57; 29.82%) and poultry (13/57; 22.81%) sources, meanwhile colistin ARGs were detected in rabbit (3), swine (3) and dairy (1) strains. 3GC resistance genes were identified in 2 beef and 1 human strains, respectively. Extended-spectrum beta-lactamase (ESBL) genes (*bla*_CTX-M1,15,55_) were rarely observed and identified in animal (2 dairy and 1 wild animal strains) and human (1) samples. One hundred and eleven different VAGs were detected among the collection. All strains carried between 1 and 37 VAGs. Virulence profile often included typical ExPEC VAGs. Notably, different pandemic (ST69, ST95, ST131) and emerging (ST10, ST23, ST58, ST117, ST405, ST648) ExPEC lineages were observed, especially in poultry meat strains (ST131, ST69, ST10, ST23, and ST117). Considering Clermont triplex PCR phylotyping method [5], the most common phylogroup identified among the collection was B1 (130; 46.6%), followed by A (81, 29%) D (47; 16.8%) and B2 (21; 7.5%).

### 4.3. Bacterial Isolation and Molecular Identification

A comprehensive description of the isolation and the molecular identification protocols are available in Massella et al., (2020) [33].

Briefly, lactose fermenting colonies were selected on MacConkey’s (Oxoid, Basington, UK) and Levine’s (Oxoid, Basington, UK) agar plates and were incubated for 18–24 h at 37 ± 1 °C. In food sample processing, an additional enrichment step in EC-Broth (Oxoid, Basington, UK) preceded strain isolation on agar plates. Gram stain and standard biochemical test (indole probe) were used for presumptive *E. coli* identification. Genomic DNA was extracted using a commercial kit (DNeasy Blood and Tissue Kit, Qiagen, Hilden, Germany), following the manufacturer’s instruction. Species identification was performed using the multiplex PCR designated by Horakova et al., (2008) [150].

### 4.4. Antimicrobial Resistance

Antimicrobial resistance phenotyping was performed with the Kirby-Bauer disk agar diffusion method in accordance with the Clinical and Laboratory Standards Institute guidelines (CLSI) [151]. The antimicrobial panel was chosen considering the antimicrobial genetic profile of the collection, the importance of antimicrobial classes in the treatment of human infections and the intrinsic resistance of *E. coli* [152]. Particular attention was focused on HP-CIAs, whose resistance is highly suspected to be linked to food-producing sector [7].

The following antimicrobials were tested: nalidixic acid (30 µg), ampicillin (10 µg) ceftazidime (10 µg), ceftiofur (30 µg), chloramphenicol (30 µg), enrofloxacin (10 µg), gentamicin (10 µg), streptomycin (10 µg), sulfisoxazole (300 µg), tetracycline (30 µg) and trimethoprim-sulfamethoxazole (25 µg).

Results were interpreted referring to the epidemiological cut-off values (ECOFFs) for *E. coli* proposed by the European Committee on Antimicrobial Susceptibility Testing (EUCAST) (http://www.eucast.org; last accessed 30 June 2020). The EUCAST clinical breakpoints for *Enterobacteriaceae* [153] or the CLSI clinical breakpoints for *Enterobacteriaceae* [151,154,155] were considered when cut-off values were not available (Supplementary Materials Table S1).

Colistin MIC was determined by the broth microdilution method using customized Sensititre™ 96-well microtitre plates (Trek Diagnostic Systems, East Grinstead, UK). Following manufacturer instructions, 10 µl of bacterial suspension (0.5 McFarland) were placed in 11 mL Mueller-Hinton Broth cation adjusted (Oxoid, Basington, UK). Fifty microliters of the final suspension were put into all wells of the same strip within 30 min after its preparation. Plates were incubated for 18–24 h at 35 °C. Strains were considered susceptible/resistant considering EUCAST ECOFF (2 mg/L) for *E. coli* (http://www.eucast.org; last accessed 30 June 2020).

The combination disk test [152] was implemented for the evaluation of ESBL producing *E. coli*. Briefly, the strains were tested for cephalosporins (ceftazidime and cefuroxime) alone and in combination with clavulanic acid, performing disk agar diffusion method. When an increase ≥5 mm in zone diameter was observed in the presence of clavulanic acid compared with the cephalosporin alone, the strain was considered an ESBL producer.

*E. coli* ATCC 25,922 was used as control strain for antimicrobial testing and as negative control for the evaluation of ESBL profile.

Strains were considered multidrug resistant (MDR) when showing resistance to three or more antimicrobial classes [156].

Lastly, we evaluated concordance between *E. coli* phenotypic AMR pattern and genotypic AMR profile, identified in the previous publication [33].

### 4.5. Phylogrouping

Phylogroup evaluation was performed using the quadruplex PCR and primers described by Clermont et al., (2013) [108], able to identify 7 different phylogroups (A, B1, B2, C, D, E and F) and cryptic clade I. Strains generically designated as cryptic clade members in the quadruplex PCR were then screened to establish the specific cryptic clade (II-III-IV-V), according to Clermont et al., (2011) method [157].

Finally, results were compared to phylogroup distribution obtained with in silico phylotyping technique reported in the previous publication [33].

### 4.6. Expec Virulence Potential

Evaluation of ExPEC virulence potential considered different epidemiological traits, such as ST and virulence gene profile (both identified in the previous study) and phylogroup belonging (according to revisited Clermont scheme) [6]. The collection was divided into 3 main groups according to ST epidemiological role in ExPEC infections [158]: the first was characterised by pandemic ExPEC STs; the second consisted of emerging ExPEC STs; the third included the remaining lineages not specifically associated to ExPEC pathogens. Attention was focused on B2 and D strains, as these phylogroups have been historically associated to ExPEC pathogens [108].

Genes coding for typical ExPEC virulence factors (VFs) of 5 functional categories (adhesins, invasins, iron acquisition systems, toxins and protectins) [11] were selected from the original virulence profile. Additional genes were also included, such as ones coding for vacuolating autotransporter toxin (*vat*), genetic island associated with newborn meningitis (*gimB*) [125] and uropathogenic specific protein (*usp*) [159] (Table 6).

The resulting ExPEC genetic profile, associated to a specific phylogroup and ST belonging, was then considered in order to establish ExPEC pathogenic potential of the collection. Association of virulence and important AMR traits (MDR, HP-CIA resistance, ESBL profile) was also investigated.

### 4.7. Statistical Analysis

Differences in the occurrence of resistance to antimicrobial agents in *E. coli* strains among different sources were tested by Pearson’s chi-square. The same statistical test was used to assert differences in phylogroup distribution among *E. coli* of different niches and association between specific resistance and phylogroup. A *p* value < 0.05 was considered statistically significant.

Diversities in phylogroup assignment in terms of number and phylotype among *E. coli* of different origin were calculated using Shannon–Weaver diversity index (H′), H′ = −∑^s^*_i_* = 1*P_i_* ln(*P_i_*), where *P_i_* is the percentage of strains belonging to the *i*-th phylotype of the total number of strains surveyed [163], and Simpson’s diversity index (D′), D *=* −∑^s^*_i_* = 1*N_i_* (*N_i_* − 1)/*N* (*N* − 1), where *N_i_* is the number of strains in the *i*-th phylotype and *N* is the total number of strains [163].

## 5. Conclusions

In conclusion, our study provides an important overview of phenotypic AMR, ExPEC virulence potential (according to the revisited Clermont scheme) and phylogroup distribution in commensal *E. coli* of animal, food and human origin.

AMR pattern in human, companion animal and most FPA categories reflected general phenotypic resistance trends and antimicrobial stewardship in Europe. Identification of human and animal (livestock and companion animal) AMR profiles in niches with a rare (fishery, mollusc) or absent (vegetable, wild animal, wild boar) direct exposure to antimicrobials suggested widespread environmental AMR pollution. Some sources (wild animal and wild boar) may represent important AMR source sentinel. AMR was mainly associated to FPAs, already suspected to play a major role in AMR diffusion.

The important virulence profile identified among the collection proposed commensal *E. coli* as ExPEC VAG source, from which potential pathogen may acquire new virulence traits. Identification of different pandemic/emerging ExPEC STs and similarities in virulence profile between commensal and clinical human ExPEC lineages could suggest ExPEC virulence potential of commensal bacteria.

Rabbit and poultry were the most concerning sources, associated to the highest AMR among all the collection and suggested as potential AMR reservoirs. Additionally, different pandemic/emerging ExPEC STs and important virulence profiles were observed in poultry strains, as already described in the literature. In poultry and rabbit sources, identification of concerning AMR and virulence profile in an important number of meat origin strains suggested the food chain as a feasible transmission path of potential multiresistant pathogens to human.

Phylogrouping revealed a complex phylotype distribution, attributed to different host and geographical factors. General concordance was observed between phenotypic and genetic AMR profile, as between molecular and in silico and phylogrouping, suggesting the revisited Clermont method as a reliable and cheaper phylotyping technique alternative.

## Figures and Tables

**Table 1:** Number of processed samples and number of isolated strains in the different sources considered in the study.

Source	Number of Samples	Number of Isolated *E. coli*	*E. coli* Occurrence (%)
Beef	33	21	63.64
Wild Boar	31	22	70.97
Vegetable	164	24	14.63
Fishery	94	24	25.53
Companion Animal	12	12	100.00
Swine	6	6	100.00
Poultry	33	25	75.76
Rabbit	14	10	71.43
Human	27	25	92.59

**Table 2:** AMR resistance rate to the molecules tested and ESBL profile among the sources investigated.

												HP-CIA			
Sources	n	R ≥ 1	MDR	CN	S	C	ST	SXT	TE	NA	ENR	EFT	CAZ	COL	ESBL
Dairy	25	6 (24%)	6 (24%)	1 (4%)	6 (24%)	3 (12%)	6 (24%)	6 (24%)	6 (24%)	3 (12%)	3 (12%)	2 (8%)	2 (8%)	1 (4%)	2 (8%)
Beef	24	7 (29.17%)	4 (16.66%)	2 (8.33%)	5 (20.83%)	2 (8.33%)	4 (16.67%)	4 (16.67%)	3 (12.5%)	1 (4.17%)	1 (4.17%)	2 (8.33%)	2 (8.33%)	0 (0%)	0 (0%)
Wild boar	22	1 (4.55%)	1 (4.55%)	0 (0%)	1 (4.55%)	0 (0%)	1 (4.55%)	1 (4.55%)	1 (4.55%)	1 (4.55%)	1 (4.55%)	0 (0%)	0 (0%)	0 (0%)	0 (0%)
Vegetable	24	2 (8.33%)	2 (8.33%)	0 (0%)	2 (8.33%)	0 (0%)	1 (4.17%)	1 (4.17%)	1 (4.55%)	0 (0%)	0 (0%)	0 (0%)	0 (0%)	0 (0%)	0 (0%)
Fishery	24	5 (20.83%)	3 (12.5%)	1 (4.17%)	3 (12.5%)	0 (0%)	2 (8.33%)	3 (12.5%)	3 (12.5%)	0 (0%)	0 (0%)	0 (0%)	0 (0%)	0 (0%)	0 (0%)
Companion animal	12	4 (33.33%)	3 (25%)	0 (0%)	1 (8.33%)	1 (8.33%)	3 (25%)	3 (25%)	4 (33.33%)	1 (8.33%)	1 (8.33%)	0 (0%)	0 (0%)	0 (0%)	0 (0%)
Swine	25	16 (64%)	6 (24%)	0 (0%)	8 (32%)	2 (8%)	5 (20%)	5 (20%)	10 (40%)	3 (12%)	3 (12%)	0 (0%)	0 (0%)	2 (8%)	0 (0%)
Poultry	25	23 (92%)	16 (64%)	1 (4%)	13 (52%)	8 (32%)	14 (56%)	15 (60%)	13 (52%)	13 (52%)	11 (44%)	0 (0%)	0 (0%)	0 (0%)	0 (0%)
Rabbit	23	23 (100%)	22 (95.65%)	6 (26.09%)	22 (95.65%)	3 (13.04%)	22 (95.65%)	22 (95.65%)	22 (95.65%)	15 (65.22%)	13 (56.52%)	0 (0%)	0 (0%)	3 (13.04%)	0 (0%)
Mollusc	25	4 (16%)	3 (12%)	0 (0%)	2 (8%)	0 (0%)	1 (4%)	1 (4%)	2 (8%)	1 (4%)	1 (4%)	0 (0%)	0 (0%)	0 (0%)	0 (0%)
Human	25	9 (36%)	7 (28%)	0 (0%)	5 (20%)	3 (12%)	6 (24%)	6 (24%)	6 (24%)	6 (24%)	6 (24%)	1 (4%)	1 (4%)	0 (0%)	1 (4%)
Wild animal	25	7 (28%)	6 (24%)	0 (0%)	3 (12%)	1 (4%)	6 (24%)	6 (24%)	5 (20%)	4 (16%)	1 (4%)	1 (4%)	1 (4%)	0 (0%)	1 (4%)
Total	279	107 (38.35%)	79 (28.32%)	11 (3.94%)	71 (25.45%)	23 (8.24%)	71 (25.45)	73 (26.16%)	76 (27.24%)	48 (17.20%)	44 (15.77%)	6 (2.15%)	6 (2.15%)	6 (2.15%)	4 (1.43%)

R ≥ 1: resistant to at least one antimicrobial; MDR: multiresistant isolate; CN: gentamicin; S: streptomycin; C: chloramphenicol; ST: sulfisoxazole; SXT: trimethoprim/sulfamethoxazole; TE: tetracycline; NA: nalidixic acid; ENR: enrofloxacin; EFT: ceftiofur; CAZ: ceftazidime; COL: colistin; ESBL: extended spectrum beta lactamase profile; HP-CIA: highest priority critically important antimicrobial.

**Table 3:** Concordance between phenotypic and genetic AMR profile identified among the collection.

Phenotypic Resistance	n	Genetic Determinants
Beta-lactams		
amipicillin	60	*bla*SHV73, *bla*TEM-1b (2)
		*bla*TEM-1a (4)
		*bla*TEM-1b (38)
		*bla*TEM-1c (8)
		*bla*TEM-1d (4)
		*bla*TEM-214 (1)
		*bla*TEM-220 (3)
ampicillin, ceftiofur, ceftazidime	6	*ampC*^#^ (2)
		*bla*CMY-2, *bla*CTX-M55, *bla*TEM-1b (1) (1)
		*bla*CTX-M1 (1)
		*bla*CTX-M1, *bla*TEM-1b (1)
		*bla*CTX-M15, *bla*TEM-1b (1)
Chloramphenicol	23	*catA1* (8)
		*catA1*,*cmlA1* (2)
		*catA2* (1)
		*cmlA1* (11)
		*mdfA*, *acrAB-TolC* (1)
Aminoglycosides		
gentamicin	1	*aac(3)_IIa* (1)
streptomycin	61	*aadA1* (16)
		*aadA2* (1)
		*aadA2b* (1)
		*strA* (2)
		*strB*(1)
		*aadA1*, *aadA2* (2)
		*strA*, *strB* (20)
		*aadA1*, *aadA2b* (6)
		*strA*, *strB*, *aadA5* (2)
		*aadA1*, *aadA2b*, *strB* (1)
		*aadA1*, *strA*, *strB* (7)
		*aadA1*, *aadA2b*, *strA*, *strB* (2)
gentamicin, streptomycin	10	*aac(3)_IIa*, *aadA1*, *aadA2b*, *strA*, *strB* (1)
		*aac(3)_IIa*, *aadA1*, *strA*, *strB* (1)
		*ant2_Ia*, *aadA1* (1)
		*aac(3)-IId*, *aadA5*, *strA*, *strB* (2)
		*aac(3)_IIa*, *aac3_IV*, *aadA1*, *aadA2b*, *strA*, *strB* (1)
		*aac(3)_IV*, *strA*, *strB* (1)
		*aac(3)_IV*, *aadA1*, *strA*, *strB* (2)
		*aac)(3)_IIa*, *strA*, *strB* (1)
Sulphonamides		
sulfisoxazole, sulfametoxazole/trimethoprim	71	*sul1* (2)
		*sul1*, *dfrA1* (13)
		*sul1*, *dfrA1*2 (1)
		*sul2* (17)
		*sul2*, *dfrA1* (3)
		*sul2*, *dfrA14* (6)
		*sul2*, *dfrA17* (1)
		*sul1*, *sul2* (1)
		*sul1*, *sul2*, *dfrA1* (7)
		*sul1*, *sul2*, *dfrA17* (3)
		*sul1*, *sul2*, *dfrA7* (1)
		*sul1*, *sul2*, *dfrA1*, *dfrA14* (1)
		*sul1*, *sul2*, *dfrA1*, *dfrA7* (1)
		*sul3* (2)
		*sul2*, *sul3*, *dfrA14* (2)
		*sul3*, *dfrA1* (3)
		*sul3*, *dfrA1*2 (2)
		*sul3*, *dfrA14* (1)
		*sul1*, *sul2*, *sul3*, *dfrA1* (2)
		*sul1*, *sul3*, *dfrA1* (2)
Sulfamethoxazole/trimethoprim	2	*dfrA1* (1)
		*dfrA1*2 (1)
Tetracycline	76	*tetA* (54)
		*tetB* (19)
		*tetA*, *tetM* (1)
		*tetA*, *tetB* (2)
Colistin	4	*mcr1* (4)
	1 *	*pmrB*^#^ (1)
ESBL profile	4	*bla*CTX-M1 (2)
		*bla*CTX-M15 (1)
		*bla*CTX-M55 (1)
(Fluoro)quinolones		
nalidixic acid	4	*gyrA*^#^ (D87G) (3)
		*gyrA*^#^ (A84P), *parC* ^#^ (S57T) (1)
nalidixic acid, enrofloxacin	45	*gyrA*^#^ (S83L) (21)
		
		*gyrA*^#^ (S83L-D87N), *parC* ^#^ (S80I) (13)
		*gyrA*^#^ (S83L-D87N), *parC* ^#^ (S80I-E84G) (2)
		*gyrA*^#^ (S83L-D87N), *parC* ^#^ (S80I-E84A) (1)
		*gyrA*^#^ (S83L-D87N), *parC* ^#^ (S80I-E84G-A56T) (1)
		*gyrA*^#^ (S83L-D87N), *parC* ^#^ (S80I), *pare* ^#^ (S458A) (3)
		*gyrA*^#^ (S83L-D87N), *parC* ^#^ (S80I), *pare* ^#^ (L416F) (1)
		*gyrA*^#^ (S83L-D87Y), *parC* ^#^ (S80I) (1)
		*gyrA*^#^ (S83L-D87Y), *parC* ^#^ (S80I), *parE*^#^ (S458A) (1)
		*qnrB19*, *pare*^#^ (I355T) (1)
		*pare*^#^ (I355T) (5)
	4 *	*parC*^#^ (A56T)(1)
		*qnrS1* (3)

^#^: chromosomal mutation; *: presence of genetic virulence determinant not associated to the expected phenotypic resistance.

**Table 4:** Representation of phylogroup distribution, Shannon Index (H′) and Simposon index (D) among the sources investigated.

Source	n	A	B1	B2	C	D	E	F	unknown	H′	D
Dairy	25	8 (32%)	9 (36%)	2 (8%)	4 (16%)	1 (4%)	1 (4%)	0 (0%)	0 (0%)	1.485	0.763
Beef	24	0 (0%)	22 (91.67%)	0 (0%)	1 (4.17%)	1 (4.17%)	0 (0%)	0 (0%)	0 (0%)	0.345	0.163
Wild boar	22	0(0%)	3 (13.64%)	6 (27.27%)	0(0%)	0 (0%)	11 (50%)	0 (0%)	2 (9.09%)	0.973	0.68
Vegetable	24	1(4.17%)	14 (58.33%)	1 (4.17%)	6 (25%)	0 (0%)	1 (4.17%)	0 (0%)	1 (4.17%)	1.191	0.616
Fishery	24	10 (41.67%)	4 (16.67%)	2 (8.33%)	5 (20.83%)	0 (0%)	3 (12.50%)	0 (0%)	0 (0%)	1.457	0.764
Companion animal	12	0 (0%)	6 (50%)	2 (16.67%)	2 (16.67%)	0 (0%)	2 (16.67%)	0 (0%)	0 (0%)	1.089	0.644
Swine	25	8 (32%)	8 (32%)	0 (0%)	8 (32%)	0 (0%)	1 (4%)	0 (0%)	0 (0%)	1.223	0.720
Poultry	25	2 (8%)	6 (24%)	1 (4%)	8 (32%)	0 (0%)	2 (8%)	5 (20%)	1 (4%)	1.691	0.817
Rabbit	23	0 (0%)	20 (86.96%)	1 (4.35%)	0 (0%)	2 (8.70%)	0 (0%)	0 (0%)	0 (0%)	0.470	0.245
Mollusc	25	2 (8%)	15 (60%)	0 (0%)	4 (16%)	3 (12%)	1 (4%)	0 (0%)	0 (0%)	1.185	0.617
Human	25	6 (24%)	2 (8%)	2 (8%)	3 (12%)	2 (8%)	3 (12%)	7 (28%)	0 (0%)	1.814	0.850
Wild animal	25	1 (4%)	18 (72%)	3 (12%)	2 (8%)	0 (0%)	0 (0%)	1 (4%)	0 (0%)	0.951	0.477
Total	279	38 (13.62%)	127 (45.52%)	20 (7.17%)	43 (15.41%)	9 (3.23%)	25 (8.96%)	13 (4.66%)	4 (1.43%)		

**Table 5:** Association between pandemic (ST69/69*, ST95, ST131), emerging (ST10/ST10*, ST23, ST58, ST117, ST405, ST648), uncommon (the remaining ones) ExPEC lineages and functional category profile identified among the collection.

Lineages	n	Functional Category Profile
Pandemic ExPEC	13	adhesin, iron acquisiton system (2)
		adhesin, iron acquisiton system, protectin (8)
		adhesin, toxin, iron acquisition system, protectin, invasin (3)
Emerging ExPEC	37	adhesin (2)
		iron acquisition system (1)
		adhesin, protectin (11)
		adhesin, iron acquisition system, protectin (18)
		adhesin, iron acquisition system, toxin, protectin (5)
Uncommon ExPEC	229	adhesin (68)
		protectin (4)
		adhesin, invasin (1)
		adhesin, toxin (3)
		adhesin, protectin (68)
		adhesin, iron acquisition system (9)
		protectin, iron acquisition system (1)
		adhesin, toxin, protectin (2)
		adhesin, iron acquisition system, protectin (50)
		adhesin, invasin, iron acquisition system, toxin (1)
		adhesin, invasin, iron acquisition system, protectin (2)
		adhesin, toxin, iron acquisition, system, protectin (12)
		adhesin, toxin, iron acquisition system, protectin, invasin (8)

**Table 6:** Representation of virulence genes identified in the collection and related virulence factors/functional categories. Each VAG is associated to specific ExPEC pathovars, where they have been commonly identified according to Kaper et al., (2004) [1], Sarowska et al., (2019) [124], Kudinha et al., (2012) [160] (*bmaE*), Tóth et al., (2009) [161] (*cdtB*), Ostblom et al., (2011) [162] (*malX*) and Schierack et al., (2008) [125] (*gimB*).

Functional Category	Virulence Factor	Gene	Pathotype
Adhesin	M-agglutinin subunit	*bmaE*	UPEC
	type 1 fimbrial adhesin	*fimH*	UPEC, NMEC, SEPEC, APEC
	iron-regulated-gene-homologue adhesin	*iha*	UPEC
	pilus associated with pyelonephritis	*papC*	UPEC, SEPEC, APEC
	pilus associated with pyelonephritis	*papG_II; papG_III*	UPEC, SEPEC, APEC
	S fimbrial adhesin	*sfaS*	UPEC, NMEC
	bifunctional enterobactin receptor/adhesin	*iha*	UPEC, NMEC
	temperature sensitive hemagglutinin	*tsh*	UPEC, NMEC, SEPEC, APEC
Inasin	invasion of brain endothelium	*ibeA*	NMEC, SEPEC, APEC
	genetic island associated with newborn meningitis	*gimB*	NMEC
Iron acquisiton system	iron-responsive element	*ireA*	UPEC
	catecholate siderophore receptor	*iroN*	UPEC, NMEC, SEPEC APEC
	ferric yersinia uptake	*fyuA*	UPEC, NMEC
	iron repressible protein	*irp2*	NMEC
	l-lysine 6 monooxigenase	*iucD*	UPEC, APEC
	ferric aerobactin receptor precursor	*iutA*	UPEC, NMEC
	periplasmic iron binding protein	*sitA*	UPEC, APEC
Toxin	cytolethal distending toxin B	*cdtB*	UPEC, NMEC, SEPEC APEC
	cytotoxic necrotising factor	*cnf1*	UPEC, MNEC, SEPEC
	haemolysin E	*hlyE*	UPEC
	serine protease autotransporters	*pic*	UPEC
	serine protease autotransporters	*sat*	UPEC
	uropathogenic specific protein	*usp*	UPEC
	vacuolating autotransporter toxin	*vat*	UPEC, APEC
Protectin	increased serum survival	*iss*	NMEC, SEPEC, APEC
	structural component of colicin V operon	*cvaC*	NMEC, SEPEC, APEC
	group II capsule antigens	*kpsMT_II*	NMEC, SEPEC
	outer membrane protein	*ompT*	UPEC, NMEC
	transfer protein	*traT*	NMEC, SEPEC
	K1 capsular polysaccharide	*neuC*	NMEC, UPEC
Other	pathogenicity-associated island marker	*malX*	UPEC, NMEC, SEPEC, APEC

## Data Availability

All relevant data is contained within the article. All datasets generated for this study are included in the article/Supplementary Materials.

## Supplementary Materials

The following are available online at https://www.mdpi.com/article/10.3390/antibiotics10040351/s1, Table S1: AMR breakpoint and cut-off considered in the present study; Table S2: phenotypic AMR profile of the collection; Table S3: genetic AMR profile of the collection; Table S4: genetic virulence profile of the collection.
